# Supplementary material for: Polarons shape the interlayer exciton emission of MoSe2/WSe2 heterobilayers
Source: Nat Commun. 2025 Oct 1;16:8735. doi: 10.1038/s41467-025-64176-6 (PMC12489084; doi:10.1038/s41467-025-64176-6)
Supplement: Supplementary file 1 — Supplementary Information [file 41467_2025_64176_MOESM1_ESM.pdf]

Supplementary Information: Polarons shape the interlayer exciton emission of $MoSe_2/WSe_2$ heterobilayers

Pedro Soubelet,[1,2] Alex Delhomme,[1] Elena Blundo,[1] Andreas V. Stier,[1] and Jonathan J. Finley[1,3]

[1] *Walter Schottky Institut and TUM School of Natural Sciences, Technische Universität München, Am Coulombwall 4, 85748 Garching, Germany.*

[2]Email: pedro.soubelet@wsi.tum.de

[3]Email: jj.finley@tum.de

(Dated: 2025-08-28)

SUPPLEMENTARY NOTE 1: SAMPLES FABRICATION AND CHARACTERIZATION

A. Samples Fabrication

Monolayer (ML) $MoSe_2$ and WSe_2 were obtained from commercial bulk crystals via mechanical exfoliation. The crystallographic directions of the 1L-TMDs were determined from the optical picture of the flakes. As the hexagonal crystal structure of a 1L-TMD lacks inversion symmetry, those crystallographic directions are defined up to a 60° rotation. Thereby, to obtain a heterobilayer (HB) with a $2H-$stacking and another with $3R-$stacking, we produced both samples from the same monolayers of $MoSe_2$ and WSe_2 with each monolayer being divided in two through the tear-and-stack method [1], as sketched in Supplementary Figure 1a. During the fabrication process, one of the resulting MLs was rotated by 180° as depicted by arrows in Supplementary Figure 1a and the micrographs in Supplementary Figure 1b and c. During the stacking of each sample, the MLs were aligned to assemble two samples with a known twist angle θ of approximately 4.5° and 57°. In $MoSe_2/WSe_2$ HBs stacked near 0°(60°), atoms within each layer form reconstructed commensurate regions [2–6]. To avoid unwanted reconstruction effects, twist angles above 2.5° and below 59° for $2H-$ and $3R-$ stacking were used, respectively [3–5]. The samples were encapsulated between thin hexagonal boron nitride (hBN) flakes using dry transfer techniques based on polycarbonate films, similar to Ref. [7]. The twist angle was confirmed by performing polarized resolved Second Harmonic Generation (SHG) experiments [8]. Supplementary Figure 1d and e, present the polar plot of the polarized resolved SHG from which the twist angle was determined.

The substrates used for these samples correspond to piezoelectric strain actuators as those used in Refs. [9] and, therefore, have evaporated gold on the surface. However, we do not present in this work any effect related with strain tuning, as well as we do not expect any effect of the gold substrate on the TMDs emission, in agreement with Ref. [10].

Supplementary Figure 1f and g present the interlayer exciton photoluminescence (PL) for the $3R-$ and the $2H-$sample, respectively. While the shape of the emission slightly depends on the position in the sample, all measurements presented in this work correspond to regions in which the emission was spectrally sharp and homogeneous. We observed that, in all such regions, the tendencies and features observed and presented are consistent.

B. Optical selection rules.

In $MoSe_2/WSe_2$ HBs, the $3R-$stacking locates the WSe_2 $K(K')$-valley at the same point in k-space as the $MoSe_2$ $K(K')$-valley, and the $2H-$stacking aligns the WSe_2 $K(K')$-valley with the $MoSe_2$ $K'(K)$-valley. However, the optical selection rules are governed not only by the valley and spin degrees of freedom but also by the Bloch phase factor within the three-fold rotational symmetry $\hat{C}_3$ [11–13]. The band alignment of the $3R$ and $2H$ HBs are illustrated in the Supplementary Figure 2a and b, respectively. Additionally, the sketches include the exciton complexes observed in this work that were also previously reported in the literature. These are a singlet interlayer exciton (IX_s) in both kinds of twist and the triplet interlayer exciton (IX_t) in the $2H$ case [12, 14].

Supplementary Figure 2c shows in the upper panels the circularly co- and cross-polarized PL for the samples stacked near $3R-$ and $2H-$configuration at left and right panels, respectively. These measurements were performed with a CW 700 nm laser and an excitation power (P_{ex}) of 40 μW. Below the PL spectra, the lower panels of Supplementary Figure 2c show the circular polarization degree, defined as $\eta = (I^+ - I^-)/(I^+ + I^-)$, where $I^+(I^-)$ is the PL intensity in the co-(cross-)polarized experiments. Although we are not using the exact $3R-$ and $2H-$stacking, the sample stacked at 4.5°(57°) exhibits circularly cross-polarized(co-polarized) photoluminescence (PL), consistent with an interlayer emission from moiré sites in samples with $3R-(2H-)$type structures [11, 12, 14–17].

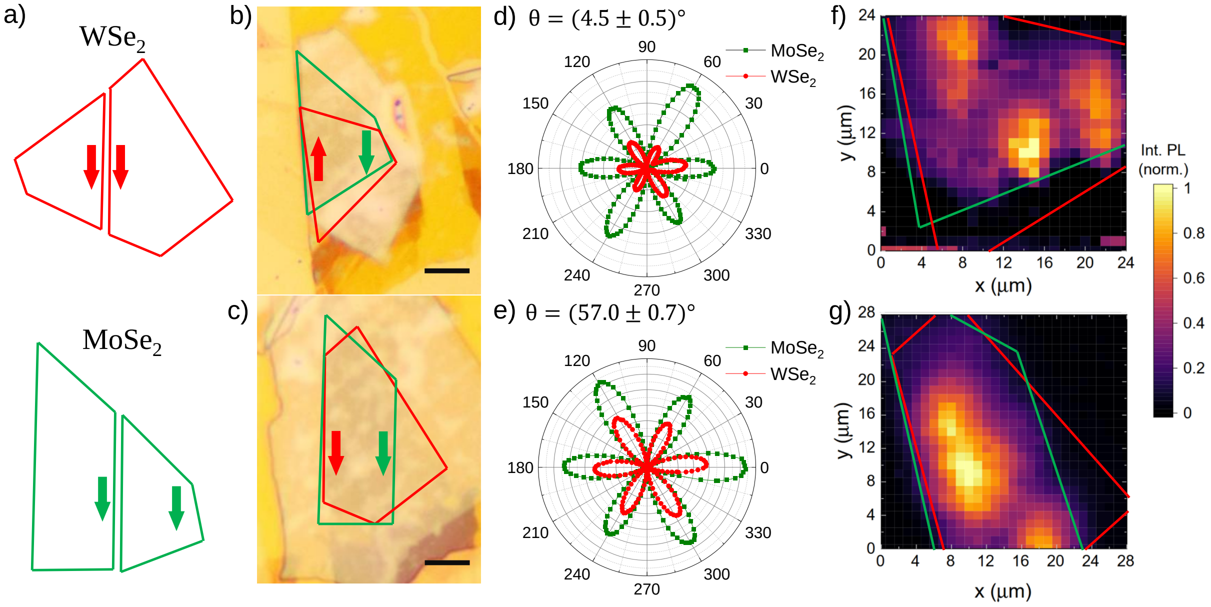

Supplementary Figure 1. **a)** Sketch of the WSe_2 (red) and $MoSe_2$ (green) monolayer and the site in which they were cut during the tear-and-stack process. **b)** and **c)** Optical micrographs of the HBs with near $3R-$ and $2H-$ stacking, respectively. Green(red) lines indicate the $MoSe_2$(WSe_2) monolayer and the scale bar correspond to $8\,\mu$m. Arrows in **a**, **b** and **c** depict the orientation of the flakes. **d)** and **e)** Polar plot of the polarization-resolved SHG intensity in the monolayer region measured for each sample. The relative angle between lobes in **d** suggest a twist angle of $(4.5 \pm 0.5)^{\circ}$ for the sample in **b** and the plot in **e** a twist angle of $(57.1 \pm 0.7)^{\circ}$ for the sample in **c**. **f)** and **g)** Integrated PL map in the spectral range of the interlayer exciton emission. for the samples presented in **b** and **c**, respectively.

SUPPLEMENTARY NOTE 2: PLE EXPERIMENTS AT DIFFERENT EXCITATION POWER

Supplementary Figure 3 displays the PLE experiments for the $3R-$ and $2H-$sample, respectively. Each figure compares the experiments performed at $P_{ex} = 1\,\mu$W and $P_{ex} = 10\,\mu$W. In this range of energies, while the sharp peaks are clearly distinguished, the observations are independent of P_{ex} and are the same as those discussed in the main text.

SUPPLEMENTARY NOTE 3: CHARACTERIZATION OF THE PHONON REPLICAS

C. Additional information regarding the normal distribution of peaks in the PL

Supplementary Figure 4a and b present, for the $3R$ and the $2H$ HBs, respectively, the PL spectra taken at different positions of each sample. While the overall emission lineshape varies along the sample, the narrow lines keep the spacing of $\sim$0.8 meV (see Supplementary Fig. 4c and d) showing that the phonon mode involved in the emission is unaffected. The presence of higher disorder in the lattice is then observed by the broadening of those lines and not by the peaks separation. For instance, the third site of the $3R-$sample displays a PL in which the peaks are comparatively much broader and are only distinguishable as shoulders in the overall emission.

It is important to note that all experiments presented in the main text were performed on the first and second spot on each sample and, for this reason, the Zero Phonon Line (ZPL) was observed in PLE, temperature dependent PL and power dependent PL. For this reason, the ZPL of the third spot on each sample is not properly defined. In those cases the peak marked as ZPL is merely an estimate.

D. Additional information regarding electron-phonon coupling factor

The phonon-assisted PL equations can be solved analytically to calculate the steady-state intensity of phonon replicas of arbitrary order [18, 19]. However, this theoretical description does not fully describe our case, since it

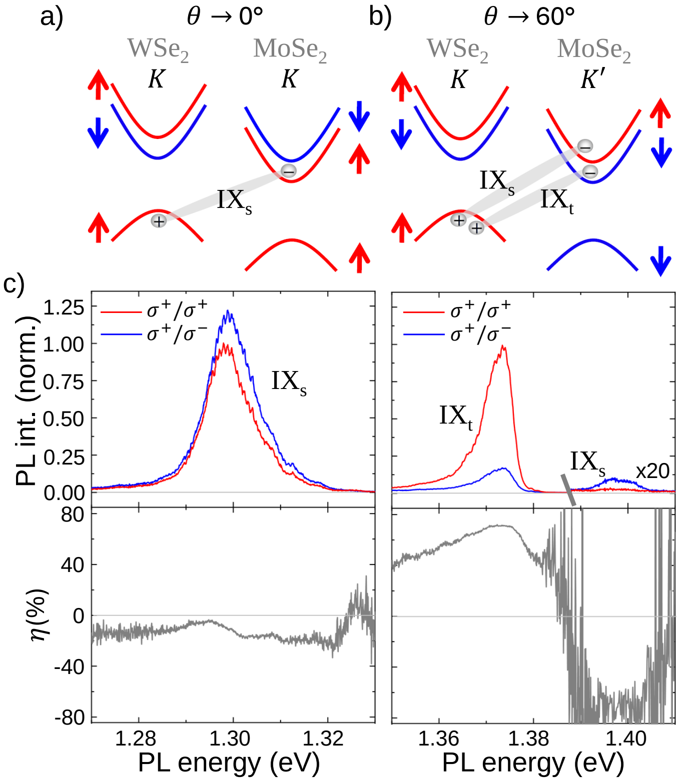

Supplementary Figure 2. **a)** and **b)** Schematics of the spin-valley configuration for samples with twist angle $\theta \rightarrow 0^{\circ}$ and $\theta \rightarrow 60^{\circ}$, respectively, the corresponding exciton complexes are plot over the electronic dispersion of each sample. **c)** Polarized resolved interlayer exciton PL (top) and circular polarization degree (bottom) for the samples stacked at 4.5° (left) and 57° (right), respectively. The spectra are normalized to the maximum intensity of the co-polarized PL in each case.

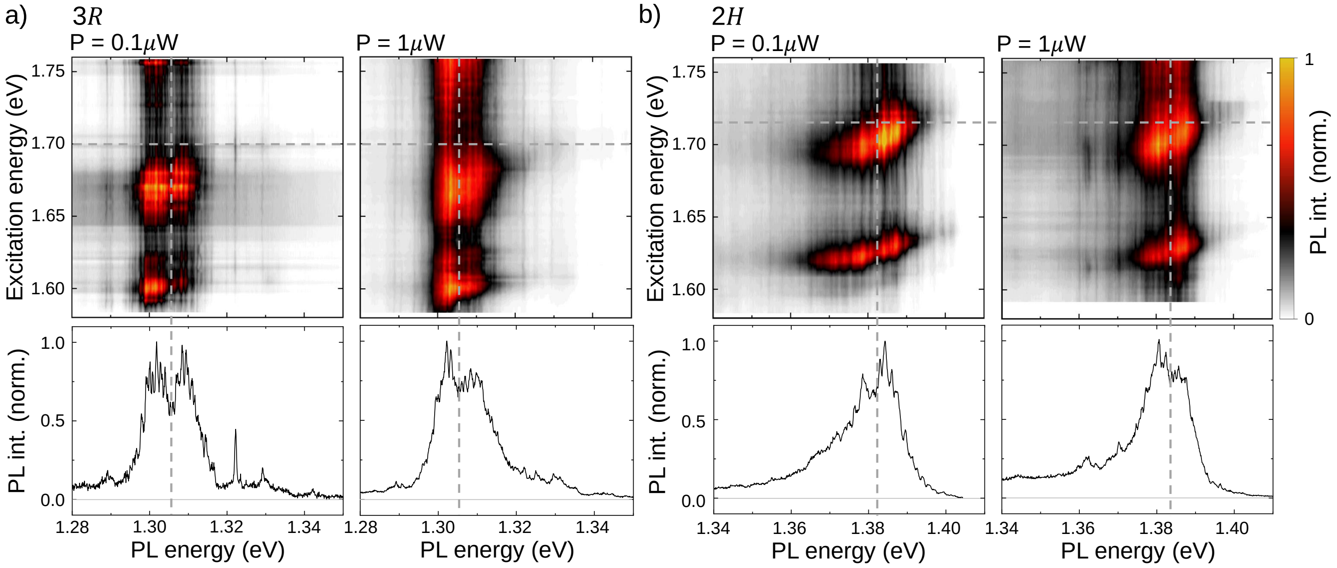

Supplementary Figure 3. **a)** and **b)** PLE experiments at different excitation power for the sample stacked in $3R$ and $2H$ configuration, respectively. On left and right panels are the experiments performed at $P_{ex} = 0.1\,\mu$W and $P_{ex} = 1\,\mu$W, respectively. Top panels: False color map of the IX emission as function of E_{ex}. The horizontal dotted line marks the selected spectra displayed in the bottom panels. Vertical dotted lines mark the ZPL.

considers a phonon thermal bath that can be neglected, resulting in a red-shifted phonon sideband and no phonon

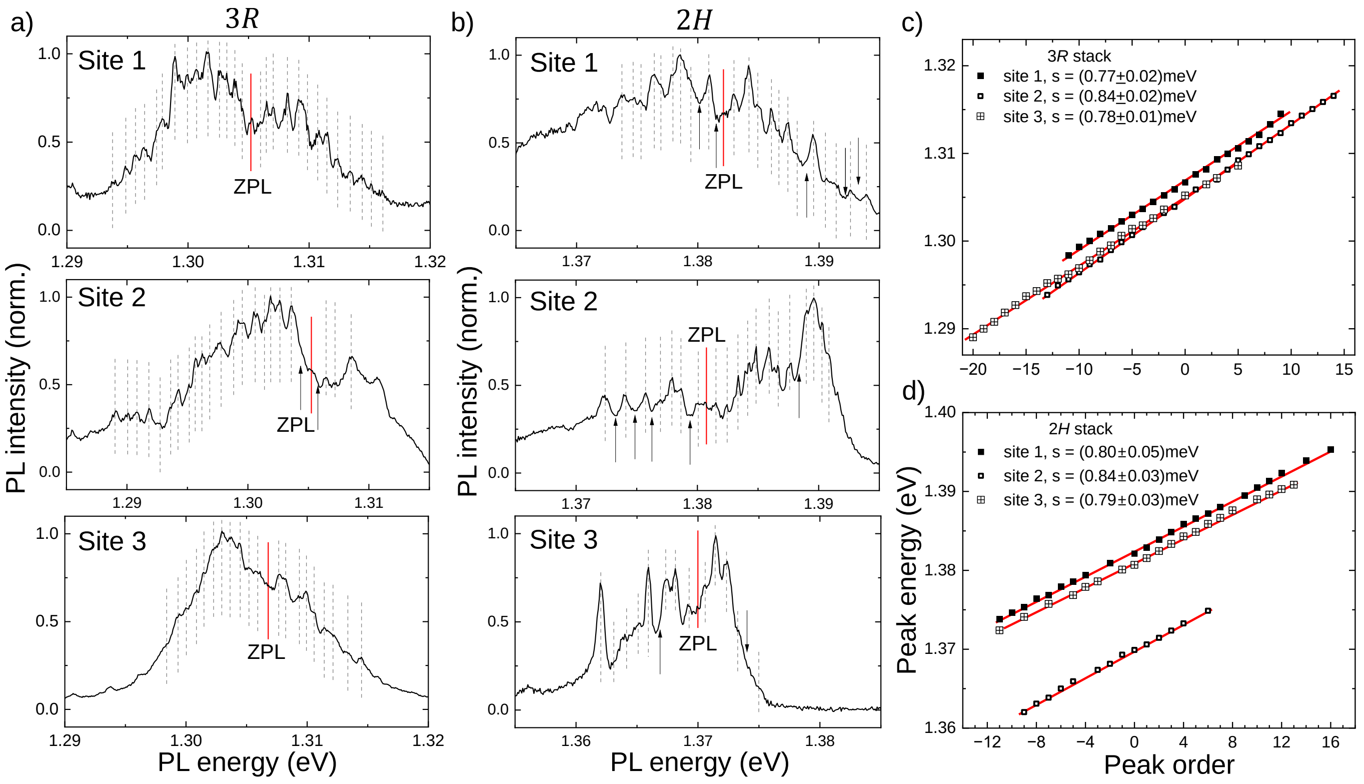

Supplementary Figure 4. **a)** and **b)** present the interlayer exciton emission in three different spots on each sample, $3R-$ and $2H-$sample, respectively. On each panel, the emmission peaks are marked with dotted vertical lines and the zero order with a red line. **c)** and **d)** Peak energy as function of peak order for the spectra in **a)** and **b)**, respectively.

absorption processes. In our case, the electron hole coupling produces a cascade process, emitting phonons to relax the excess energy from the *intra*-layer exciton energy to the IX state. Therefore, not only the thermal energy is higher than the phonons involved in the e-p coupling, but also phonon absorptions that lead to a blueshifted sideband must be considered.

Polaron spectra are frequently described through a summation of Lorentzian profiles of the form

$$\sum_N = \frac{A_N \left(\frac{\Gamma_N}{2}\right)^2}{(\omega - \omega_N)^2 + \left(\frac{\Gamma_N}{2}\right)^2}, \tag{1}$$

where ω_N is the central frequency of the Nth peak and Γ_N is its linewidth. In the strong coupling regime [20, 21], the individual peak intensity A_N is well described by a Poisson distribution function

$$A_N = A_0 \frac{e^{-\alpha_{H-R}} \alpha_{H-R}^N}{N!} \tag{2}$$

where α_{H-R} is the Huang-Rhys factor that characterizes the strength of the e-ph coupling and A_0 is the peak intensity of the ZPL. Note that the Poisson distribution considers only phonon emission processes and phonon absorptions are not included in the description. For this reason, each exciton-phonon interaction results in a redshift of the polaron emission. In our case, the intensity of the Nth emission line is composed by absorption and emission processes, i.e., $N = \alpha - \beta$, where $\alpha(\beta)$ corresponds to the number of absorbed(emitted) phonons [18]. Therefore, equation 2 may not reproduce our observations. However, we estimate the Huang-Rhys factor of each sample by fitting expression 1 to the spectra from Fig.2b of the main text and then calculate α_{H-R} through expression 2.

Supplementary Figure 4a and b presents, for the $3R-$ and the $2H-$sample, respectively, the fitting of the spectra with multiple Lorentzian curves. Note that in our case, it presents some difficulties because the distance between phonon replicas is similar to their linewidth. The peaks used for the calculation are marked with a gray rectangular shape and correspond to the redshifted phonon sideband. Supplementary Figure 4c and d display the extracted peak intensities and the extracted maximum peak intensity with black and gray squares from the emission phonon sideband. The gray line displays the fitted Poisson distribution curve resulting in a Huang-Rhys factor of 3.1 ± 0.4 and 1.5 ± 0.2

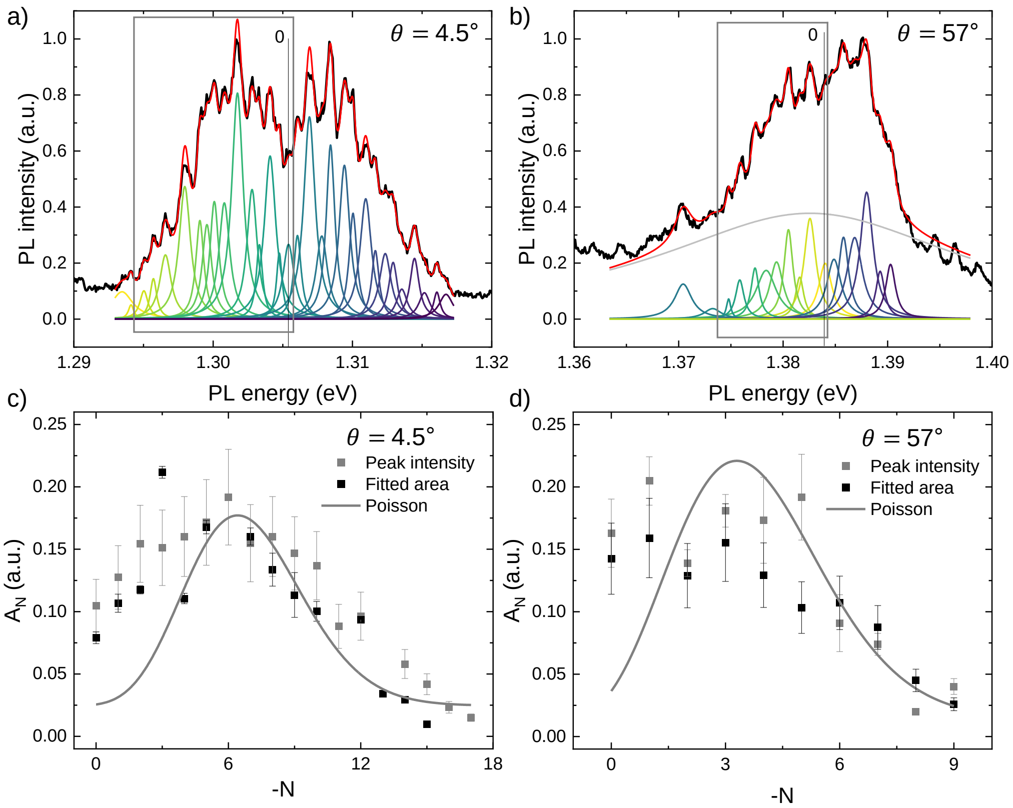

Supplementary Figure 5. **a)** and **b)** PL spectra for the $3R$ and $2H$ samples, respectively. On each spectra, the fitting of Lorentzian curves is presented in a colour scheme varying from yellow to violet. The experiments are in black and the fitting is superimposed in red. The grey curve in the $2H$ sample correspond to the background. **c)** and **d)** Extracted intensities (gray squares) and fitted area (black squares) of the peaks marked with a rectangle in figure a and b. In gray, is the Poisson distribution fitting that results in a Huang-Rhys factor of 3.1 ± 0.4 and 1.6 ± 0.2 for the $3R-$ and the $2H-$sample, respectively.

for the $3R-$ and the $2H-$sample, respectively. Note that in expression 2 α is the e-ph coupling in three dimensions, we have scaled it by a factor of $3\pi/4$ for 2D systems [22, 23].

Note that the HB intensity distribution is broader than the Poisson distribution fitting. This difference may result from the absorption processes that are not included in the theoretical description.

SUPPLEMENTARY NOTE 4: ADDITIONAL INFORMATION REGARDING THE TEMPERATURE SERIES

In this section we complement the information regarding the temperature dependent PL presented in the main text by adding the data corresponding to the $3R-$sample. The experiments were performed with a CW laser, $P_{ex} = 300\,\mathrm{nW}$ and $E_{exc} = 1.96\,\mathrm{eV}$. The spectra, normalized to their maximum intensity at 7 K, are presented in Supplementary Figure 6, the left(right) panel presents the data for the $3R-(2H-)$sample. The grey dotted lines mark the position of the ZPL. Both samples display the same behaviour; at low temperature high and low energy sidebands show similar intensity but, by increasing the temperature, the high energy sideband is strongly suppressed. The narrow emission lines progressively fade out as the temperature increases and, above 20 K for the high energy sideband and 30 K for the lower one, they are almost indistinguishable.

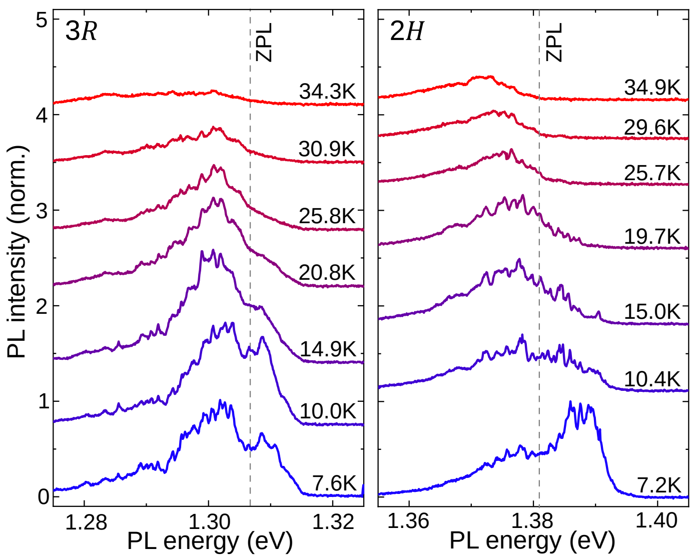

Supplementary Figure 6. Temperature dependent PL spectra for the $3R-$sample (left) and the $2H-$sample (right).

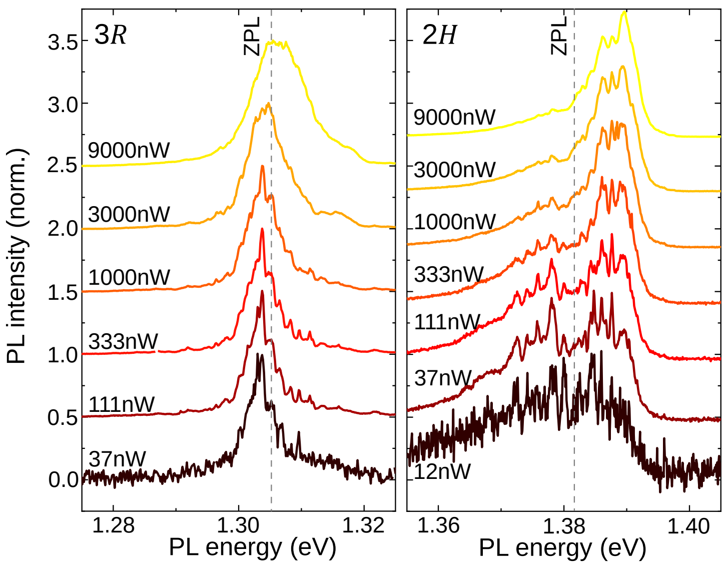

Supplementary Figure 7. Power dependent PL spectra for the $3R-$sample (left) and the $2H-$sample (right).

SUPPLEMENTARY NOTE 5: ADDITIONAL INFORMATION REGARDING THE POWER SERIES

E. Experiments

In this section, we complement the information regarding the power dependent experiments shown in the main text by adding the data of the sample stacked at $4.5°$. In both samples, the experiments were performed at $7\,\mathrm{K}$, with a CW laser at $E_{exc} = 1.96\,\mathrm{eV}$. Supplementary Figure 7 shows the power dependent PL, where the left(right) panel corresponds to the $3R-(2H-)$sample. In both cases, the distinct emission peaks coalesce into a broad emission line as P_{ex} increases. Additionally, there is a clear blueshift of the overall emission by increasing P_{ex}, in agreement with previous reports [14, 17, 24, 25]. However, a careful observation of Supplementary Figure 7 reveals that the observed blueshift is due to a variation in the relative intensities between the low and high energy sidebands.

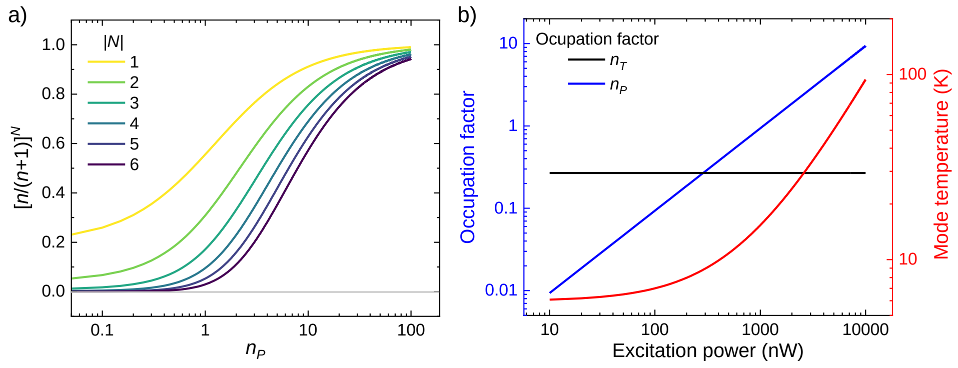

Supplementary Figure 8. **a)** $\frac{\tau_{N<0}}{\tau_{N>0}}\left(\frac{n_T+n_P}{n_T+n_P+1}\right)^N$ considering $n_T = 0.25$ and n_P varying from 0 to 100 for some $|N|$ varying from 1 to 6. **b)** Thermal and power dependent occupation factors and mode temperature derived from the n_P and proportionality constant derived in the main text.

SUPPLEMENTARY NOTE 6: PHENOMENOLOGICAL DESCRIPTION

We describe the power dependent PL with a phenomenological model that accounts for phonon replicas of arbitrary order under the following assumptions: i) The phonons that contribute to the sidebands are generated during the charge transfer and energy relaxation processes. Consequently, the phonon occupation number is $n = n_T + n_P$, where n_T is the thermal occupation and n_P are the optically generated phonons, ii) The Nth emission line corresponds to $N-$ phonon absorption (for $N > 0$) and $N-$ phonon emission (for $N < 0$). These approximations allow us to determine the relative intensity between the low and high energy sideband. It is well established in rate equation models that the emission intensity of a particular excited level is proportional to $1/\tau$, where τ is the radiative lifetime of the transition. On the other hand, as the peaks we describe are phonon replicas of the ZPL, their abundance is a function of the phonon occupation factor. Following the peak labeling of the main text, $N > 0$ are IX emission in which the IX is dressed with N phonons and its intensity in PL experiments is n^N/τ_N. In the case $N < 0$, however, there is always the possibility of spontaneous emission and, therefore, the intensity of a phonon emission replica is $(n+1)^{-N}/\tau_N$. The ratio between positive and negative phonon replicas of the same order is then

$$\frac{I_{N>0}}{I_{N<0}} = \frac{\tau_{N<0}}{\tau_{N>0}}\left(\frac{n}{n+1}\right)^N = \frac{\tau_{N<0}}{\tau_{N>0}}\left(\frac{n_T+n_P}{n_T+n_P+1}\right)^N. \tag{3}$$

Consequently, the limit $n_P \to 0$ becomes

$$\lim_{n_P\to 0}\frac{I_{N>0}}{I_{N<0}} = \frac{\tau_{N<0}}{\tau_{N>0}}\left(\frac{n_T}{n_T+1}\right)^N \tag{4}$$

and the limit $n_P \to \infty$

$$\lim_{n_P\to\infty}\frac{I_{N>0}}{I_{N<0}} = \frac{\tau_{N<0}}{\tau_{N>0}}. \tag{5}$$

Supplementary Figure 8a shows for some Ns the function $\frac{\tau_{N<0}}{\tau_{N>0}}\left(\frac{n_T+n_P}{n_T+n_P+1}\right)^N$ considering $n_T = 0.25$ and n_P varying from 0 to 100.

In the main text, we did not make any distinction between modes of different order and only considered the difference between high and low energy sideband. As result, we obtained an average behaviour of the different phonon replicas that, as expressed by eq. 5, allowed us to estimate the ratio between the low and high energy sideband lifetime.

Supplementary Figure 8b presents, considering the parameters obtained in the main text, i.e. the average behaviour of eq. 3, the comparison between the thermal and power dependent occupation factor. The right axis displays the mode temperature, showing that at a relative low P_{exc} the mode temperature is much higher than that of the lattice ($\sim 7\,$K).

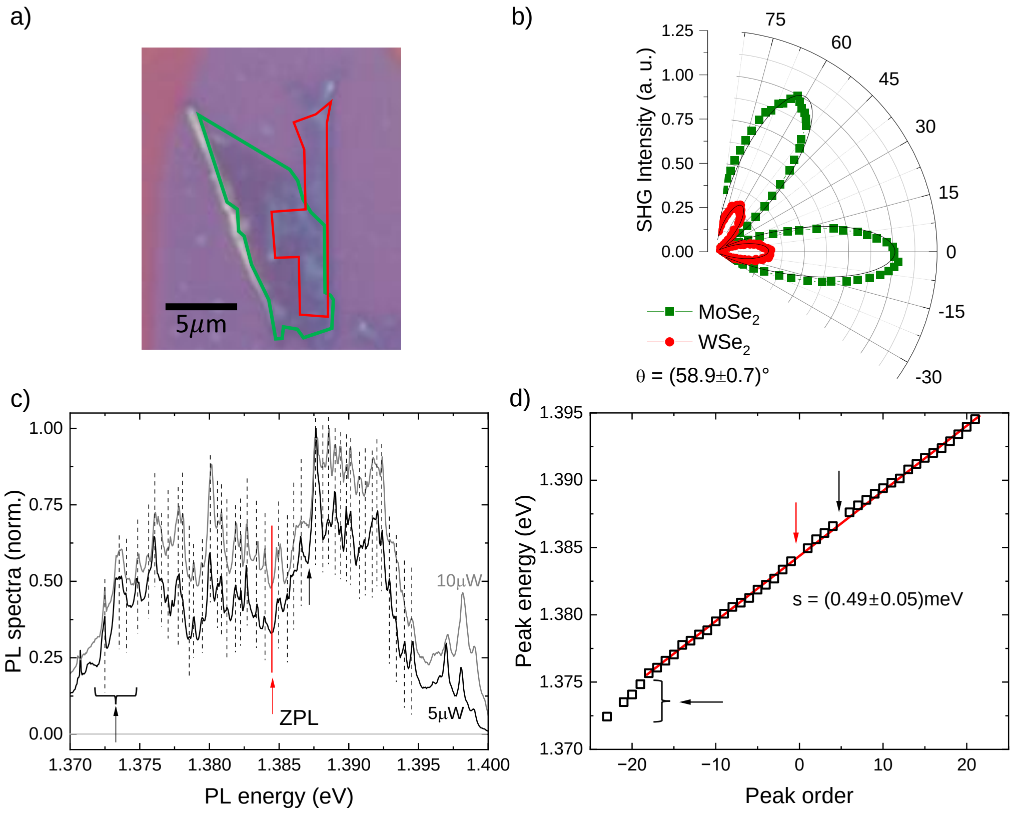

Supplementary Figure 9. **a)** Optical micrographs of the additional HB with near $2H-$ stacking. Green(red) lines indicate the 1L-$MoSe_2$(1L-WSe_2). **b)** Polar plot of the polarization-resolved SHG intensity in the monolayer region measured for each sample. The relative angle between lobes suggests a twist angle of $(58.9 \pm 0.7)^\circ$. **c)** Interlayer exciton emission at two different P_{ex}. The emission peaks are marked with dotted vertical lines and the zero order with a red line. The arrows mark emission lines that lack or spectral regions in which the lines are not evenly spaced. **d)** Peak energy as a function of peak order for the spectra in **c**.

SUPPLEMENTARY NOTE 7: ADDITIONAL SAMPLE $\theta \simeq 1^\circ$

To explore how the polaron emission bands depend on the twist angle in $MoSe_2/WSe_2$ HBs, we fabricated an additional sample. Increasing the twist angle above 6°(below 55°) leads to greater momentum mismatch between the electron and hole comprising the IX, leading to a HB emission up to three order of magnitude dimmer than the emission of samples with twist angles near 0°(60°) [17, 26]. As it makes it challenging to resolve the narrow emission lines, we stacked a third sample with a twist angle near 60° (or, equivalently, 0°). Supplementary Figure 9a presents an optical image of this sample, which was transfered onto a 270 nm-thick SiO_2/Si substrate. Polarization-resolved SHG measurements [8] and PL spectroscopy confirmed a twist angle of $(58.9 \pm 0.7)^\circ$, as shown in Supplementary Fig. 9b and c, respectively.

Photoluminescence spectra of the sample, presented in Supplementary Fig. 9c, were acquired with 5 μW and 10 μW CW laser excitation at 720 nm, resonant with the WSe_2 A-exciton. The spectra are qualitatively identical to those discussed in the main text, featuring double lobed broad emission and a sequence of narrow emission lines. A comparative analysis of the two spectra allows us to extract the peak distribution, shown in Supplementary Fig. 9d. Arrows in Supplementary Figs. 9c and d indicate emission lines that are absent or spectral regions where lines deviate from the uniform spacing. Despite minor irregularities, the emission lines exhibit an approximately uniform energy spacing of $\sim$0.5 meV. This value is significantly smaller compared to the peak spacing observed in the samples with the larger twist angles, suggesting a subtle dependence of the polaron features on θ and highlighting the need for further understanding of the phonon mode that leads to the polaron formation.

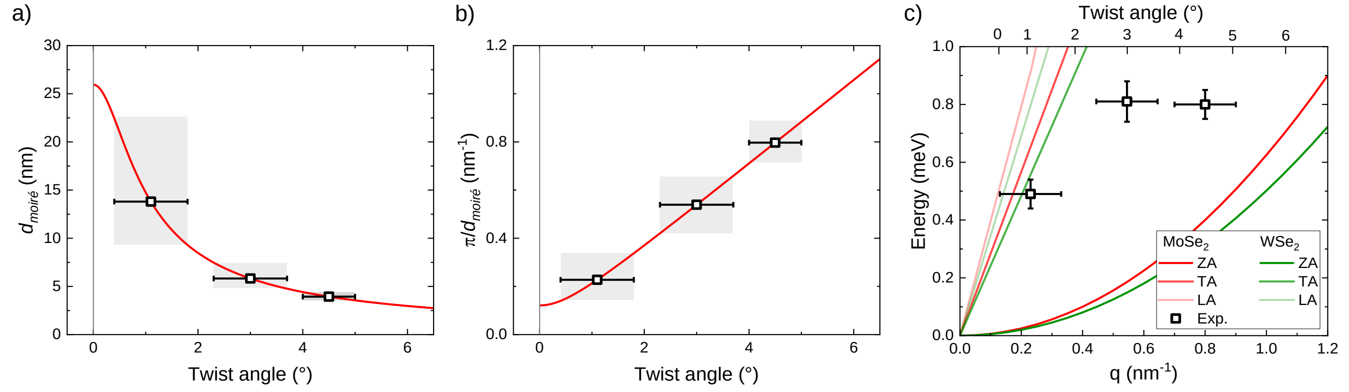

Supplementary Figure 10. **a)** Moiré lattice parameter as a function of twist angle for a $MoSe_2/WSe_2$ HB. **b)** Edge of the moiré mini-Brillouin zone as a function of twist angle for a $MoSe_2/WSe_2$ HB. Dots in **a** and **b** correspond to the investigated samples. The rectangular shades highlight the uncertainty derived from the twist angle error bar. **c)** Acoustic phonon dispersion for $MoSe_2$ and WSe_2 extracted from Ref.[44]. The dots superimposed on the solid lines correspond to the investigated samples. The position along the x axis of each dot is the calculated edge of the respective mini-Brillouin zone.

SUPPLEMENTARY NOTE 8: PHONON MODES IMPLICATED IN THE IX-POLARONS FORMATION

In solid-state hosts, various vibrational modes can contribute to the emergence of phonon sidebands. In pristine semiconductors, these vibrational modes are usually lattice phonons [18, 19, 23, 27]. The coupling between a phonon mode and an exciton state is generally inferred from the mode atomic displacement and wavelength, which modulate the exciton wavefunction, in conjunction with the vibrational density of states at the relevant energy [23, 27].

On the other hand, systems containing optically active defects, such as color centers in diamond [28, 29], can also exhibit pronounced phonon sidebands. Here, the defects induce significant distortions in the lattice crystal, giving rise to local and quasi-local vibrational modes, which are detached from the bulk phonon dispersion. In such cases, these modes exist solely due to the presence of the impurity, and their frequencies are determined not by the crystal phonon dispersion, but rather by the mass of the impurity and the local interatomic bonding forces [29]. In our case, this hypothesis suggests that the Coulomb interaction between the electron and hole that comprise the moiré-localized IX significantly perturbs the lattice, generating phonon modes that are decoupled from the lattice phonon dispersion. These local distortions could, therefore, facilitate the formation of energetically discrete and spatially localized vibrational modes that are intrinsic to the polaronic state.

In 2D materials, the unambiguous identification of the specific phonon mode involved in polaron formation is often insufficiently defined [23, 27, 30]. While theoretical ab-initio calculations provide the material phonon dispersion, in practice, a variety of extrinsic and intrinsic factors can affect the real phonon dispersion. Notably, phonon renormalization in 2D materials is strongly dependent on the number of layers [31, 32], doping level [33], applied strain [34], nanostructured environment [35] and temperature [36]. Additionally, TMD HBs are subject to reconstruction phenomena [37], heterostrain [38] and the hybridization of vibrational states between the 1L-TMDs and with the rest of the heterostructure [39], factors that are inherently difficult to control and quantify. This complexity increases further in the case of IX-polarons [40] since the phonon modes involved in the IX-polaron formation are difficult to observe them due to their low energy.

In this section we discuss the nature of the phonon modes that take part in the IX-polarons. The observed phonon energies of $\sim 0.5\,\text{meV}$ to $\sim 0.8\,\text{meV}$ are smaller than the interlayer breathing mode energy in $MoSe_2/WSe_2$ HBs, which is $\sim$3.3 meV [41, 42]. Therefore, if the vibrational modes belong to the 2D dispersion, they would be situated within the acoustic phonon branches, in particularly the ZA branch as proposed in Ref. [40, 43]. Furthermore, the phonon involved in the polaron formation must be located at the edge of the mini-Brillouin zone ($\pi/d_{moiré}$) to absorb the momentum mismatch of the IX relative to the light cone (see Fig.1b in the main text). Here, the folded phonon dispersion flattens towards $\pi/d_{moiré}$, exhibiting a maximum in the phonon density of states that enhance the e-ph interaction probability.

Supplementary Figure 10a shows the calculated moiré lattice parameter $d_{moiré}$ as a function of the twist angle for $MoSe_2/WSe_2$ HBs, calculated following Ref. [38] with a lattice parameter of $a_{MoSe_2} = 0.329\,\text{nm}$ for the 1L-$MoSe_2$ [45] and 0.4% smaller for the 1L-WSe_2 [45, 46]. The three dots correspond to the samples presented in this work. Note that the moiré lattice parameter is insensitive to the lack of inversion symmetry in the 1L-TMDs and, therefore, the sample stacked at $57°(58.9°)$ is, from the moiré lattice point of view, equivalent to a sample stacked at $3°(1.1°)$. The rectangular shades in Supplementary Fig. 10a highlight the uncertainty derived from the twist angle error bar. While

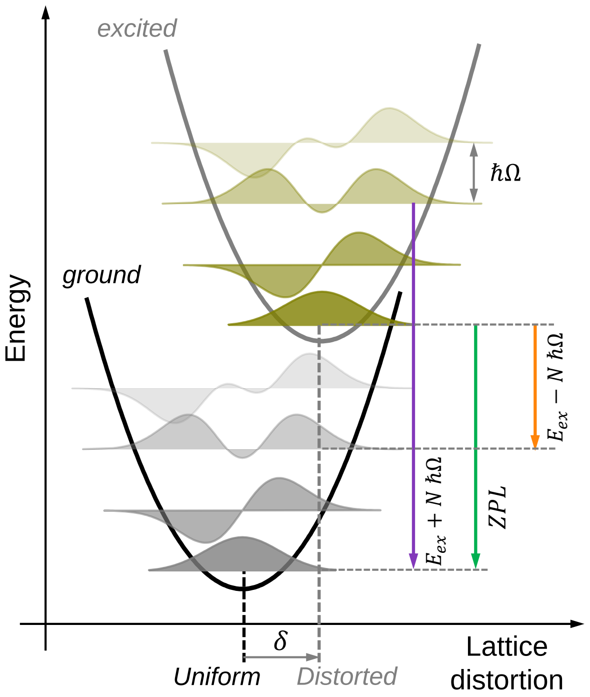

Supplementary Figure 11. Configuration coordinate diagram illustrating the potential energy surfaces of the ground and excited states of a crystal lattice containing an IX, plotted as a function of a generalized lattice distortion coordinate. The two potentials are offset by a displacement parameter δ, representing the shift in equilibrium lattice configuration between electronic states. Quantized vibrational levels within each potential are separated by the phonon energy $\hbar\Omega$. The vertical optical transition between the lowest vibrational levels defines the ZPL at energy E_{ex}, while transitions involving additional phonon emission or absorption give rise to redshifted ($E_{ex} - N\hbar\Omega$) and blueshifted ($E_{ex} + N\hbar\Omega$) phonon sidebands, respectively.

the two samples from the main text show an approximately similar $d_{moiré}$ (6 nm and 4.5 nm), the additional sample in the SN 7 displays a much larger $d_{moiré}$ of 14 nm.

Supplementary Figure 10b shows the calculated edge of the mini-Brillouin zone ($\pi/d_{moiré}$) as a function of twist angle, the dots correspond to the specific samples studied in this work. Lastly, Supplementary Fig. 10c shows the acoustic phonon dispersion relations for $MoSe_2$ and WSe_2 from Ref.[44]. The phonon dispersion is plotted in the region of small energies and wavevectors, with the energy and $\pi/d_{moiré}$ for the studied samples superimposed. While the sample stacked at 58.9° aligns within experimental errors with the TA and LA branches of the 1L-TMD materials, the samples with the larger twist angles clearly exhibit phonon modes that do not correspond to any vibrational branch. Although the previously mentioned renormalization processes may modify the phonon dispersion, this mismatch suggests that the vibrational modes involved in the polaron formation do not belong to the intrinsic 2D dispersion of these materials.

The discrepancy between the phonon dispersion relation and the energy and proposed wavevector of the observed phonon modes suggests a scalable reorganization of the monolayer crystal upon IX formation, leading to the emergence of localized vibrations that subsequently take part in the polaron formation. This process is schematically illustrated in Supplementary Fig. 11, using a configurational coordinate diagram usually employed to present the Franck–Condon principle. Upon IX formation, the lattice undergoes a dynamic distortion which lowers the energy of the system. Thus, the IX is surrounded by the localized phonon cloud forming the polaronic state. As described in the main text and SN 6, the phonon is a low energy mode whose occupation number n is greater than or equal to the thermal occupation number n_T. Therefore, during the HB emission process, the IX emits or absorbs phonons forming the emission phonon sidebands.

Within such a picture, the twist angle dependence observed in the phonon energy is then a measure of the crystal deformation within a moiré minimum in which an IX is confined. Consequentially, the samples shown in the main text, with similar moiré lattice parameter display a similar phonon energy. On the other hand, the sample presented in the SN 7 host IXs in a larger moiré unit cell that requires a smaller crystal deformation, i.e., it requires a lower energy phonon to form polarons (see Supplementary Fig.10a and c).

REFERENCES

[1] K. Kim, M. Yankowitz, B. Fallahazad, S. Kang, H. C. Movva, S. Huang, S. Larentis, C. M. Corbet, T. Taniguchi, K. Watanabe et al., "Van der Waals heterostructures with high accuracy rotational alignment", Nano Letters **16**, 1989–1995 (2016).

[2] C. Woods, L. Britnell, A. Eckmann, R. Ma, J. Lu, H. Guo, X. Lin, G. Yu, Y. Cao, R. V. Gorbachev et al., "Commensurate–incommensurate transition in graphene on hexagonal boron nitride", Nature Physics **10**, 451–456 (2014).

[3] A. Weston, Y. Zou, V. Enaldiev, A. Summerfield, N. Clark, V. Zólyomi, A. Graham, C. Yelgel, S. Magorrian, M. Zhou et al., "Atomic reconstruction in twisted bilayers of transition metal dichalcogenides", Nature Nanotechnology **15**, 592–597 (2020).

[4] T. I. Andersen, G. Scuri, A. Sushko, K. De Greve, J. Sung, Y. Zhou, D. S. Wild, R. J. Gelly, H. Heo, D. Bérubé et al., "Excitons in a reconstructed moiré potential in twisted WSe_2/WSe_2 homobilayers", Nature Materials **20**, 480–487 (2021).

[5] V. Enaldiev, V. Zolyomi, C. Yelgel, S. Magorrian and V. Fal'Ko, "Stacking domains and dislocation networks in marginally twisted bilayers of transition metal dichalcogenides", Physical Review Letters **124**, 206101 (2020).

[6] D. Edelberg, H. Kumar, V. Shenoy, H. Ochoa and A. N. Pasupathy, "Tunable strain soliton networks confine electrons in van der Waals materials", Nature Physics **16**, 1097–1102 (2020).

[7] A. Castellanos-Gomez, M. Buscema, R. Molenaar, V. Singh, L. Janssen, H. S. Van Der Zant and G. A. Steele, "Deterministic transfer of two-dimensional materials by all-dry viscoelastic stamping", 2D Materials **1**, 011002 (2014).

[8] W.-T. Hsu, Z.-A. Zhao, L.-J. Li, C.-H. Chen, M.-H. Chiu, P.-S. Chang, Y.-C. Chou and W.-H. Chang, "Second harmonic generation from artificially stacked transition metal dichalcogenide twisted bilayers", ACS nano **8**, 2951–2958 (2014).

[9] Z. An, P. Soubelet, Y. Zhumagulov, M. Zopf, A. Delhomme, C. Qian, P. E. Faria Junior, J. Fabian, X. Cao, J. Yang, A. V. Stier, F. Ding and J. J. Finley, "Strain control of exciton and trion spin-valley dynamics in monolayer transition metal dichalcogenides", Phys. Rev. B **108**, L041404 (2023).

[10] I. Paradisanos, G. Wang, E. M. Alexeev, A. R. Cadore, X. Marie, A. C. Ferrari, M. M. Glazov and B. Urbaszek, "Efficient phonon cascades in WSe_2 monolayers", Nature Communications **12**, 538 (2021).

[11] H. Yu, G.-B. Liu and W. Yao, "Brightened spin-triplet interlayer excitons and optical selection rules in van der Waals heterobilayers", 2D Materials **5**, 035021 (2018).

[12] K. Shinokita, K. Watanabe, T. Taniguchi and K. Matsuda, "Valley relaxation of the moiré excitons in a $WSe_2/MoSe_2$ heterobilayer", ACS nano **16**, 16862–16868 (2022).

[13] T. Wang, S. Miao, Z. Li, Y. Meng, Z. Lu, Z. Lian, M. Blei, T. Taniguchi, K. Watanabe, S. Tongay et al., "Giant valley-Zeeman splitting from spin-singlet and spin-triplet interlayer excitons in $WSe_2/MoSe_2$ heterostructure", Nano Letters **20**, 694–700 (2019).

[14] M. Brotons-Gisbert, H. Baek, A. Campbell, K. Watanabe, T. Taniguchi and B. D. Gerardot, "Moiré-trapped interlayer trions in a charge-tunable $WSe_2/MoSe_2$ heterobilayer", Physical Review X **11**, 031033 (2021).

[15] F. Mahdikhanysarvejahany, D. N. Shanks, M. Klein, Q. Wang, M. R. Koehler, D. G. Mandrus, T. Taniguchi, K. Watanabe, O. L. Monti, B. J. LeRoy et al., "Localized interlayer excitons in $MoSe_2$–WSe_2 heterostructures without a moiré potential", Nature Communications **13**, 5354 (2022).

[16] H. Yu, G.-B. Liu, J. Tang, X. Xu and W. Yao, "Moiré excitons: from programmable quantum emitter arrays to spin-orbit–coupled artificial lattices", Science advances **3**, e1701696 (2017).

[17] K. L. Seyler, P. Rivera, H. Yu, N. P. Wilson, E. L. Ray, D. G. Mandrus, J. Yan, W. Yao and X. Xu, "Signatures of moiré-trapped valley excitons in $MoSe_2/WSe_2$ heterobilayers", Nature **567**, 66–70 (2019).

[18] T. Feldtmann, M. Kira and S. W. Koch, "Phonon sidebands in semiconductor luminescence", physica status solidi (b) **246**, 332–336 (2009).

[19] T. Feldtmann, M. Kira and S. W. Koch, "Theoretical analysis of higher-order phonon sidebands in semiconductor luminescence spectra", Journal of luminescence **130**, 107–113 (2010).

[20] D. C. Langreth, "Singularities in the x-ray spectra of metals", Physical Review B **1**, 471 (1970).

[21] M. de Jong, L. Seijo, A. Meijerink and F. T. Rabouw, "Resolving the ambiguity in the relation between Stokes shift and Huang–Rhys parameter", Physical Chemistry Chemical Physics **17**, 16959–16969 (2015).

[22] F. Peeters and J. Devreese, "Scaling relations between the two-and three-dimensional polarons for static and dynamical properties", Physical Review B **36**, 4442 (1987).

[23] W. Jin, H. H. Kim, Z. Ye, G. Ye, L. Rojas, X. Luo, B. Yang, F. Yin, J. S. A. Horng, S. Tian et al., "Observation of the polaronic character of excitons in a two-dimensional semiconducting magnet CrI_3", Nature Communications **11**, 4780 (2020).

[24] P. Nagler, G. Plechinger, M. V. Ballottin, A. Mitioglu, S. Meier, N. Paradiso, C. Strunk, A. Chernikov, P. C. Christianen, C. Schüller et al., "Interlayer exciton dynamics in a dichalcogenide monolayer heterostructure", 2D Materials **4**, 025112 (2017).

[25] W. Li, X. Lu, J. Wu and A. Srivastava, "Optical control of the valley Zeeman effect through many-exciton interactions", Nature Nanotechnology **16**, 148–152 (2021).

[26] P. K. Nayak, Y. Horbatenko, S. Ahn, G. Kim, J.-U. Lee, K. Y. Ma, A.-R. Jang, H. Lim, D. Kim, S. Ryu et al., "Probing evolution of twist-angle-dependent interlayer excitons in $MoSe_2/WSe_2$ van der Waals heterostructures", ACS nano **11**, 4041–4050 (2017).

[27] M. Kang, S. W. Jung, W. J. Shin, Y. Sohn, S. H. Ryu, T. K. Kim, M. Hoesch and K. S. Kim, "Holstein polaron in a valley-degenerate two-dimensional semiconductor", Nature Materials **17**, 676–680 (2018).

[28] R. Brout and W. Visscher, "Suggested experiment on approximate localized modes in crystals", Physical Review Letters **9**, 54 (1962).

[29] A. Zaitsev, "Vibronic spectra of impurity-related optical centers in diamond", Physical Review B **61**, 12909 (2000).

[30] M. Dyksik, D. Beret, M. Baranowski, H. Duim, S. Moyano, K. Posmyk, A. Mlayah, S. Adjokatse, D. K. Maude, M. A. Loi et al., "Polaron vibronic progression shapes the optical response of 2D perovskites", Advanced Science **11**, 2305182 (2024).

[31] X. Zhang, X.-F. Qiao, W. Shi, J.-B. Wu, D.-S. Jiang and P.-H. Tan, "Phonon and raman scattering of two-dimensional transition metal dichalcogenides from monolayer, multilayer to bulk material", Chemical Society Reviews **44**, 2757–2785 (2015).

[32] P. Soubelet, A. E. Bruchhausen, A. Fainstein, K. Nogajewski and C. Faugeras, "Resonance effects in the Raman scattering of monolayer and few-layer $MoSe_2$", Physical Review B **93**, 155407 (2016).

[33] B. Chakraborty, A. Bera, D. Muthu, S. Bhowmick, U. V. Waghmare and A. Sood, "Symmetry-dependent phonon renormalization in monolayer MoS_2 transistor", Physical Review B—Condensed Matter and Materials Physics **85**, 161403 (2012).

[34] Y. Wang, C. Cong, C. Qiu and T. Yu, "Raman spectroscopy study of lattice vibration and crystallographic orientation of monolayer MoS_2 under uniaxial strain", small **9**, 2857–2861 (2013).

[35] C. Qian, V. Villafañe, P. Soubelet, A. Hötger, T. Taniguchi, K. Watanabe, N. P. Wilson, A. V. Stier, A. W. Holleitner and J. J. Finley, "Nonlocal exciton-photon interactions in hybrid high-Q beam nanocavities with encapsulated MoS_2 monolayers", Phys. Rev. Lett. **128**, 237403 (2022).

[36] S. J. R. Tan, S. Sarkar, X. Zhao, X. Luo, Y. Z. Luo, S. M. Poh, I. Abdelwahab, W. Zhou, T. Venkatesan, W. Chen et al., "Temperature-and phase-dependent phonon renormalization in 1T-MoS_2", Acs Nano **12**, 5051–5058 (2018).

[37] J. Quan, L. Linhart, M.-L. Lin, D. Lee, J. Zhu, C.-Y. Wang, W.-T. Hsu, J. Choi, J. Embley, C. Young et al., "Phonon renormalization in reconstructed MoS_2 moiré superlattices", Nature Materials **20**, 1100–1105 (2021).

[38] M. Kögl, P. Soubelet, M. Brotons-Gisbert, A. Stier, B. Gerardot and J. Finley, "Moiré straintronics: a universal platform for reconfigurable quantum materials", npj 2D Materials and Applications **7**, 32 (2023).

[39] L. Li, J. Chen, L. Hu, Z. Qiu, Z. Zou, R. Liu, L. Zheng and C. Cong, "Moiré collective vibrations in atomically thin van der Waals superlattices", Nature Communications **16**, 4117 (2025).

[40] Z. Iakovlev, M. Semina, M. Glazov and E. Y. Sherman, "Flexural deformations and collapse of bilayer two-dimensional crystals by interlayer excitons", Physical Review B **105**, 205305 (2022).

[41] C. Li, A. V. Scherbakov, P. Soubelet, A. K. Samusev, C. Ruppert, N. Balakrishnan, V. E. Gusev, A. V. Stier, J. J. Finley, M. Bayer et al., "Coherent phonons in van der waals $MoSe_2/WSe_2$ heterobilayers", Nano Letters **23**, 8186–8193 (2023).

[42] S. Y. Lim, H.-g. Kim, Y. W. Choi, T. Taniguchi, K. Watanabe, H. J. Choi and H. Cheong, "Modulation of phonons and excitons due to moiré potentials in twisted bilayer of $WSe_2/MoSe_2$", Acs Nano **17**, 13938–13947 (2023).

[43] M. A. Semina, M. M. Glazov and E. Sherman, "Interlayer exciton–polaron in atomically thin semiconductors", Annalen der Physik **532**, 2000339 (2020).

[44] M. Zulfiqar, Y. Zhao, G. Li, Z. Li and J. Ni, "Intrinsic thermal conductivities of monolayer transition metal dichalcogenides MX2 (M= Mo, W; X= S, Se, Te)", Scientific Reports **9**, 4571 (2019).

[45] J. Kang, S. Tongay, J. Zhou, J. Li and J. Wu, "Band offsets and heterostructures of two-dimensional semiconductors", Applied Physics Letters **102** (2013).

[46] W. Schutte, J. De Boer and F. Jellinek, "Crystal structures of tungsten disulfide and diselenide", Journal of Solid State Chemistry **70**, 207–209 (1987).
